# An Essay on Curvatures and Diseases of the Spine

**Published:** 1825-01-01

**Authors:** 


*An Essay on Curvatures and Diseases of the Spine, including all the Forms of Spinal Distortion: to which the Fothergillian Gold Medal was awarded by the Medical Society of London; and presented, at a Special General Meeting, on the 3d of May,* 1824*: with some Additions.* By R. W. Bampfield, Esq. one of the Surgeons to the Royal Metropolitan Infirmary for Diseases of Children; Fellow of the

Medical Society of London; Author of an Essay on Hemeralopia, or Night Blindness; of Practical Treatises on Tropical and Scorbutic Dysentery, &c. Octavo, pp. 378. London, October 1824.

Of late years, works on the Spine have presented themselves in as regular succession as the Ghosts of Banquo. One treatise can hardly be perused before "another and another still succeeds." Pathological investigations run, like the currents of the high seas, sometimes quick, sometimes slow—never in any regular order, nor amenable to any fixed law. Affections of the spine seem to be one of the prominent studies of the day, which many have cultivated, but not with equal success:—"non ex aliquovis ligno fit mercurius." Several circumstances are necessary to a successful investigation of any medical subject. Many who have the talent, have not the opportunity—and still more have the means, but want the inclination. It is very evident that Mr. Bampfield is possessed of the requisite talent for the investigation on which he has entered, and that he has had ample field for putting his talent out to good interest, instead of hiding it in the earth, like the sluggard of old. Mr. Bampfield had his attention directed to the present subject, several years ago, in consequence of meeting with some interesting cases in private practice; but he had not sufficient field for the complete investigation of spinal diseases till he became attached to the Royal Metropolitan Infirmary for Diseases of Children, where numerous cases presented themselves, the experience derived from which is now meritoriously laid before the public at large. Previously to this, Mr. Bampfield had published some essays on the subject in the London Medical and Physical Journal, but not in a connected or arranged form. The proposal of the Fothergillian Medal, by the Medical Society of London, determined our author to arrange his facts and observations into a concise essay, which was sent to the committee in December 1823, prior to the appearance of publications on the same subject by Mr. Shaw, Dr. Dods, Dr. Jarrold, Mr. Charles Bell, and Dr. Ollivier.—The work gained the prize, and has now issued from the press, with emendations and additions.

The Essay is divided into fourteen chapters and an appendix, investigating somewhat fully and formally the various diseases of the spinal column and its important contents, together with their causes, consequences, and modes of treatment. Considering how very freely we analyzed the several works which have already appeared on the subject of spinal diseases, it will not be necessary to enter so minutely into a review of the present volume, as if it had appeared a year or two ago. Still, as the construction of the work affords us a facility of offering to our readers a kind of *resumé*, or recapitulation of what has been scattered through various numbers of this Journal, we shall skim over the different topics, and condense some of the most prominent features into the present article.

*Nosology.* Mr. Bampfield divides diseases of the spine into those chiefly affecting the bones and joints of the vertebral column, and those affecting the medulla spinalis and its coverings. Of the distortions and projections of the spine, he makes two species—one denominated curvature—the other, angular projection. Of the former, there are three varieties, namely, *excurvation*, or curvature outwards;—*incurvation*, or curvature inwards;—and *lateral*, or curvature to one or both sides. The angular projection has not any varieties.

Curvatures of the spine may take place in any part of the column, but they most commonly occur in the dorsal region, frequently including the whole of the dorsal vertebræ, in the form of an arc. The curve may be divided into two extreme points, and a centre or middle, which is its angle and the point of pressure from the superincumbent weight—a circumstance which ought to be remembered. The *angular projection* generally takes place in the lower dorsal or lumbar vertebræ, commonly involving some of the former. Curvatures and projections of the spine are generally formed during the growth of the body—from the age of 1 to 21 years. They do occur, however, though not frequently, from the age of 21 to 40. Beyond this they are very rare.

Till recently, curvature of the spine was considered as associated with caries as its cause; but more extensive investigation has proved that spinal curvatures are more frequently without than with caries of the vertebræ.

1. *Excurvation.* This is the most frequent of all spinal curvatures—occurring during childhood, puberty, and more rarely in old age—except as the natural curve of senility. The following symptomatology cannot be abridged without detriment.

"The commencement of excurvation of the spine is sometimes marked by pain of the vertebræ about to be involved in the curvature, which is more particularly felt on using exercise, in the erect posture, or may be, at all times, excited by pressure on the spinous processes; in the latter case, an adult would shrink from the pain and mention it; a child would cry. Should inflammation of any of the constituent parts of the vertebral joints exist, the pain would be of a more permanent nature or of more frequent recurrence, and the patient will be considerably relieved by assuming the horizontal posture, and in a less degree by bending the spine backwards. The majority of cases are not preceded by any pain of the vertebræ, that attracts so much attention, or occasions so much suffering as to lead to voluntary complaint. In some cases, chiefly in those accompanied with inflammation, if a sponge wrung out of hot water be passed along the spine, it will create increase of heat and pain in the part disposed to curvature, but this is not a result obtainable in the generality of cases. An adult generally states the pain to be of an obtuse kind and feeling like a weight, but it can only be inferred that a young child feels pain by its crying, when made to sit up or walk about. At this period, if the spine be accurately examined, particularly if it be bent a little forwards, some of the spinous processes of the dorsal vertebræ, which are the seat of pain, may be observed to project more irregularly than the rest, so that they stretch the skin over them, and make it appear white and shining, resembling the phenomenon of the stretched skin over the knuckles of a clenched fist; yet this appearance of the curvature at its commencement is only of a temporary nature, as by laying the patient in a proper horizontal posture, and using mechanical aid, the vertebral column can be restored to its spinal line. About the time the above evidence of incipient curvature is manifest, many adults will complain of a sense of weakness in their back, of a lassitude that disinclines them from exertion, and of fatigue being soon induced by exercise, all of which disposed them to seek repose and avoid motion. A child, if he have not previously gained the use of his legs, has the ability of using the lower limbs deferred long beyond the usual period children should walk, and if he had acquired the power of walking, he will gradually lose that remarkable spirit of activity and endless motion peculiar to children, and become listless and languid, seeking his mother's lap for a resting place. Should these symptoms of weakness and indisposition to exercise lead to the frequent adoption of the recumbent posture, the curvature will increase very slowly, or its progress may be arrested for a time. Should however, the patient not lie down more than usual, but be seated in a chair during the day, where the vertebræ will have to bear the superincumbent weight of the head, &c. especially if he walk about as much as he is able, the curvature will advance with considerable rapidity, and after a little increase. If the patient be placed on his feet, he cannot maintain the vertebral column in the erect attitude, but inclines the upper part forwards; and he moves with a slow, hesitating, tottering gait, and is soon tired, or he cannot direct his steps to the proper point, and he stumbles, trips or crosses his legs, and falls down crying. In this state a child dreads to be put down, and when on his feet will, with tears, cling to his mother or nurse for support, and if deprived of it, he will totter, his knees will bend, and he will soon fall, unless caught. The lower extremities gradually diminish in size, as the curvature advances, and frequently lose part of their natural sensibility and temperature; the muscles exchange their plump and firm feel, for a softer one, and their power daily decreases. The patient will often sit with his legs crossed, and in bed will sometimes experience pains in his knees, and convulsive actions of the muscles of the thighs, which are more particularly felt and described by the adult, whose loss of muscular power is more generally accompanied with a sense of coldness and diminished sensibility and firmness of the extremities. If a palsy of the lower limbs be now induced, there is of course an end to locomotion; the muscles and cellular texture of the extremities waste and wither; the limbs become flaccid and cold, and, if lifted up, will fall down like a mass of inanimate matter; the joints are more rigid than in ordinary paralysis, and the skin frequently retains some power of sensation." 12.

This calamitous event supervenes only occasionally; for the majority of adults and minors retain sufficient muscular power of the extremities to enable them to move about in their room, by the aid of chairs, tables, &c. If the patient be confined to the horizontal posture, more especially the facial, when the excurvation is in the incipient stage, no permanent curvature will ensue, provided the said posture be long enough employed for the dorsal muscles to recover their energy, or the disposition to inflammation and scrofulous action of the vertebræ be removed. On the other hand, should the patient incautiously continue to sit up and walk about, a curvature of the spine ensues and gradually increases. The first projection, already described, becomes accompanied by another and another, until many or the whole of the dorsal vertebræ form a curve.

"As the curvature increases, the spinous processes of the dorsal vertebræ gradually separate from each other; but the greatest degree of separation takes place about the middle of the curve, where the space of half an inch is left between them, and the forefinger can be easily inserted. When they are separated at this distance, it may be always inferred with much probability, if not certainty, that the horizontal surfaces of the bodies of the vertebræ, of which these spinous processes are corresponding parts, are either greatly or wholly absorbed, as well as the intervertebral substances between them." 14.

The above process is accompanied necessarily by distortion of the ribs and sternum. The general health becomes impaired, and particular organs and functions suffer derangement. These derangements are described, with much fidelity, by Mr. Bampfield, as affecting the muscles, the bones of the spine and chest, the spinal marrow and nerves, the ligaments, the arterial and absorbent systems, the thoracic, abdominal, and pelvic viscera. We can glance but very slightly at these important topics, recommending the reader to study carefully the excellent original.

*The Muscles.* The loss of power, of density, and of firmness in the muscles of the lower extremities and of the back is generally conspicuous in this disease. This loss of power in the muscles of the back prevents the patient maintaining the vertebral column erect, for more than a few minutes at a time, and at length he is obliged to support the upper part of the body by resting with his arms or elbows on some collateral fixture. The dorsal muscles become not only elongated, from the detour which they make round the convexity, but they also appear to be diminished in size at the middle or most projecting points of the curvature—an appearance which is verified by dissection. The abdominal muscles and diaphragm also have their natural positions deranged, and their functions consequently impeded. So much weakened have the abdominal muscles become, in some cases, that Mr. Copeland has considered them to be paralytic—a state, however, in which Mr. Bampfield has never seen them. The muscles of the upper extremities do not always escape, but are not so often affected as those of the lower. In a few cases, the sphincters of the bladder and rectum lose their tone. For numerous remarks, of a highly interesting nature, respecting these muscular affections in excurvation, we must refer to Mr. Bampfield's work, from page 15 to page 22.

*Affections of the Bones.* These are considered, *first*, in respect to their distortion—and *secondly*, in respect to the disorganization, or destruction that ensues. By an ingenious chain of physiological and mechanical reasoning, Mr. Bampfield arrives at the conclusion, that, in simple cases, when the muscles of the back, from their debility, are unable to sustain the vertebral column erect by their contractile power, the top of the spine necessarily bends forward, forming a projection or excurvation backwards. The consequence is, that unnatural pressure is unavoidably made on the anterior portions of the bodies of the vertebræ and intervertebral cartilages. As the relative proportions of the vertebræ must come to be altered by this unequal pressure, so the curvature will increase—and if this take place, as it usually does, during the growth of the body, permanent curvature must be produced, bringing in its train a distortion of all the bones of the thorax.

"It might have been previously mentioned, that when the curvature is extensive and the back very weak, a slight lateral curvatnre is sometimes superadded to the excurvation, which in these cases sometimes becomes double; the lateral curvature in this instance seems to arise from the patient seeking to rest or support himself, when sitting or standing, by leaning to one side, after he finds himself weary or fatigued from inclining forward. When the double excurvation occurs, the first curve is formed by the dorsal vertebræ, and the second, which is always very slight, by the lumbar vertebræ." 28.

So much for the distortion of the bones. We next come to their destruction. This, Mr. Bampfield observes, is accomplished in two different ways—presenting after death dissimilar appearances. One mode is by caries or *ulcerative* absorption—the other by *simple* or progressive absorption. Caries of the bodies of the vertebræ is, in general, the result of that slow scrofulous inflammation which peculiarly invades the cancellous structure of these, and the articulating extremities of other bones; in which case, the remaining portions of the bodies of the vertebræ are often soft, so as to be easily divided with a common knife. Caries also ensues from common chronic inflammation, in which case, the portions of the vertebræ contiguous to the carious part are unusually vascular, and of a dark colour, but they preserve their natural texture and hardness.—The bodies of the vertebræ are destroyed in the most irregular manner, as the drawings found in works, and the specimens in anatomical collections, amply illustrate. Carious vertebræ often produce inflammation of the soft parts contiguous, terminating in suppuration.

"Hence, abscesses are formed, which first collect opposite the carious vertebræ;—if these should be the dorsal, the abscess will collect in the posterior mediastinum, and the pus may separate the pleura from its natural adhesions, and by pressing on the lungs occasion inflammation of the pleura, and distressing dyspnœa; or it may burst into the thorax and occasion death. If the lower dorsal or lumbar vertebræ be the seat of caries, the collection of pus may insinuate itself between the crura of the diaphragm, may separate the peritonæum from the diaphragm or its other natural adhesions, or it may penetrate through the peritonæum among the intestines, and excite peritonitis that may terminate in death. The collections of pus, even when as high as the upper part of the posterior mediastinum, sometimes gradually insinuate themselves along the spine, and present anteriorly in the course of the psoæ muscles or of the spermatic chord, or these abscesses may take an outward direction, and break posteriorly on one side or the other of the vertebræ, so as to become what are strictly denominated lumbar abscesses." 31.

"The destruction of the bodies of the vertebræ by progressive absorption is effected in a different manner from that by caries or ulcerative absorption. Progressive absorption is always begun and continued on their horizontal surfaces, and proceeds in an oblique direction from the anterior to the posterior parts of their bodies, so that if the disease be suffered to run its course without interruption, the bodies of the vertebræ are first reduced to a cuneiform shape, and are then rendered thinner and thinner, until they are wholly absorbed, and as this process is, in general, the consequence of unnatural pressure, the intervertebral cartilage becomes absorbed previously or contemporaneously with the bone. By this process, I have seen, on dissection, the bodies of five vertebræ entirely destroyed, and that of another partly absorbed. I have also seen the whole of the dorsal vertebræ and intervertebral cartilages reduced more or less into the cuneiform shape by this process, without the integrity of the joints being done away with. In instances of more limited disease of this nature, only two or four vertebræ are reduced to this shape. No abscesses form in cases of disorganization from the action of the absorbents. In such, it is probable, the bone is less solid than natural, or its organization so imperfect as to render it liable to absorption." 32.

The curvature is in an inverse ratio to the number of vertebræ destroyed, being more acute or projecting when there are two or three, than when there are four or five destroyed. It is curious that the posterior surfaces of the bodies of the vertebræ forming the anterior part of the spinal canal, are almost always the last to yield to, or be destroyed by, the ulcerative or progressive absorption, and generally escape so far as to leave a bony continuity to that part of the canal. Nature, however, is sometimes reduced, as it were, to the necessity of uniting several spinous processes by anchylosis, to maintain the said bony continuity, and insure a competent defence to the medulla spinalis.

The ribs become deranged in form and structure, in proportion to the extent of the excurvation. The sternum generally projects forwards. The right side of the chest is usually more malformed than the left, but the false ribs are seldom implicated.

*Affections of the Spinal Canal and Contents.* If the curvature be formed during the growth of the body, the calibre of the spinal canal becomes contracted in size, at the part where it passes through the curved vertebræ. But its interior surface is generally preserved smooth and continuous—"by such wonderful powers does the Creator protect and preserve vital parts." When the excurvation has arisen in an adult, and where the growth has been completed, the above-mentioned diminution of calibre in the spinal canal has not appeared in the dissections which Mr. Bampfield has made. The foramina on the sides of the vertebræ, for the passage of the spinal nerves, must necessarily take the situation of the vertebræ, and though occasionally diminished in size, like the part of the canal with which they communicate, he has never found them obliterated or destroyed. The anomalous symptoms caused by derangements of the spinal chord would be endless to enumerate.

"The tortuous course of the spinal chord, the compressed appearance and diminished size of a part of its length, and the longer course of the nerves, which are joined to this curved and projecting portion of it, do not uniformly produce similar effects on the system; nor does the degree of nervous power lost, necessarily depend upon the extent of the curvature: for a patient with a very small degree of curvature and with but little vertebral pain, may be affected with paralysis of the lower limbs, with general weakness and a torpor of the digestive and urinary organs, whilst another, who has had several bodies of the vertebræ destroyed, and whose dissection has disclosed a contracted spinal canal and chord, shall have been enabled to walk about to the last hour, and shall have preserved the animal functions in a state but little impaired. Yet it appears to be quite certain, that many of the symptoms which supervene in this complaint, must be attributed to the derangements of the spinal chord and nerves occasioned by the curvature. To refer to this cause only some which affect the nervous system, I might mention head-ache, fits, and every loss of sensation of parts or of muscular motion. The disordered respiration is also partly owing to the same cause. I have been induced to believe too, that inflammation and curvature of the spine predispose to inflammation of the brain, for in the year 1822, three patients who had been under my care for diseases of the spine, were attacked with phrenitis and died. The first, a boy, after having been cured of spinal inflammation; the second, a girl, during her treatment for the angular projection of the spine; and the third, a girl, after having been cured of excurvation of the spine, and sent into the country. Many affected with curvatures are subject to a variety of nervous complaints. Dissections have disclosed the trunk of the intercostal or great sympathetic nerve, to be either shortened in proportion to the length of the body diminished by the destruction of the bodies of the vertebræ or by the curvature of the spine, or bent into a circuitous course, and it is probable its shortened dimensions or tortuous course impair the power of the nerves derived from it." 44.

*Affections of the Ligaments.* Inflammation of these parts is generally propagated from, or excited by, caries in the bodies of the vertebræ and in the intervertebral substance. In either case it usually terminates in ulceration, and irregular destruction of the said ligaments. The ligamentum anticum commune and ligamenta intervertebralia are much more frequently affected than the posterior ligaments of the spine.

*Arterial and Absorbent Systems, &c.* When distortion of the spine has produced the oval conformation of the chest, the heart and its vessels are pushed from their natural situations, and the circulation is consequently deranged, whilst palpitation, in some instances, ensues, with other phenomena, inducing the erroneous supposition of organic disease of the heart.

The functions of the lungs, stomach, and other viscera are indeed often deranged, and various are the anomalous affections resulting as a consequence. Indigestion, asthma, hepatic derangement, and "an appaling catalogue of maladies," are the natural attendants on spinal deformity and disease.

*Etiology.* It has been laid down before, by our author, that spinal curvatures depend on ulceration or absorption of the vertebræ. But then we are to enquire what is the cause of this caries or absorption? Scrofula appears to be the great remote or predisposing cause, while contusions, falls, or sprains may excite the disease into action. Rickets, syphilis, rheumatism, &c. may also be enumerated among the causes of spinal disease. Here Mr. Bampfield enters into an examination of the opinions of Dr. Edward Harrison, who is well known to look upon relaxation of the ligaments as a fertile cause of spinal distortions leading, in fact, to luxation or subluxation of the vertebræ. Our author distinctly denies "that either permanent curvature of the spine, or luxation of the vertebræ, is caused by relaxation of the vertebral ligaments." His reasons for this denial are, *first*, because the joints of the spine are secured, not by one, but by many ligaments—and dissection has not disclosed to Mr. Bampfield that relaxed condition of the vertebral ligaments, which could be justly considered the cause of curvature of the spine. *Secondly*, he maintains that relaxation of ligament, if it did exist, would not cause spinal curvature, so long as the bodies of the vertebræ and the intervertebral substances preserved their natural dimensions—"and this they could and must do, if the disease be confined to relaxation of ligament." *Thirdly*, if this relaxation of ligaments were the cause, the bones ought to be as readily replaced as displaced, the same as is observed in the ankle joint. But this is not the case; for Mr. Bampfield has never seen an instance of permanent curvature of the spine, in which the projecting and arched bones could be reduced, at once, to their proper spinal line, by any force or means whatever. *Fourthly*, when several of the bodies of the vertebræ are destroyed, what powers prevent the spine from separating or dividing at this point?—The answer must be, says Mr. Bampfield, that there is no other competent power than the ligaments, assisted a little by the muscles.—*Fifthly*, if the spinal ligaments were relaxed and elongated, the practice of forcible extension of the spine by machinery would be very injudicious and pernicious, as it would tend to loosen and elongate them further—"yet the physician alluded to always employs this remedy." Mr. Bampfield therefore denies the premises and admits not the consequences—luxation and subluxation. The best and greatest authorities, Mr. Bampfield observes, declare there is no dislocation—for example Pott, Sir Astley Cooper, and Boyer.

Numerous facts, Mr. Bampfield avers, support him in stating debility of the dorsal muscles, however induced, to be the cause of temporary curvature; and, by its conducing to habitually bad positions, he considers it to be the predisposing cause of *permanent* spinal curvature. "Debility of the dorsal muscles has been induced by diseased brain, rheumatism, or the general weakness ensuing from dentition, or febrile diseases."

*Proximate Cause.* Mr. Bampfield thinks that, if an inference may be allowed from the results of cases left to nature, he would conclude, that, in curvatures of the spine, there frequently exists a predisposition to absorption, *not independent of pressure*, but which renders them liable to be absorbed from its effects. This predisposition he remarks, is probably removed, or ceases, before the vertebræ are relieved from pressure, otherwise the absorption would proceed *ad infinitum*, or at all events, until so many of the vertebral bodies, or their anterior portions were absorbed that the upper part of the sound spine could no longer come in contact with the lower. That this is not always the case is ascertained by dissection, which shews that a deposition of osseous matter, in many instances, takes place and arrests the destructive process of absorption. Add to this, that every day's experience proves that the diseased surfaces of the affected vertebræ gradually become united by anchylosis, even when the patients have declined all surgical aid, spurned all restraint, and taken no pains to relieve the vertebral column from the pressure of superincumbent weight.

"The proximate or immediate causes of permanent excurvation, as of vertebral distortion in general, should be sought for in some morbid alteration or destruction of the structural parts of the bones and intervertebral cartilages. Inflammation, ulceration, and caries, terminating in absorption and destruction of any of the bony parts of the vertebræ or intervertebral substance; progressive absorption of these parts from pressure; an increased or unequal growth either of the bodies or processes of the vertebræ, producing a disproportion of thickness and size between their relative parts, such as are seen in other bones more exposed to view; and progressive absorption or ulcerative destruction of the intervertebral cartilages, from any cause, may be considered ultimate effects, which occasion permanent distortion of the spine by mechanical agency, for they all alter the natural proportions of the spinal pyramid, on which its erect attitude principally, and of necessity depends.

"The morbid alteration most frequently met with, is a reduction of the bodies of the vertebræ and intervertebral substances from a state of equal thickness to a cuneiform shape; the ordinary destruction produces either a partial or total removal of one or many of those bodies and intervertebral substances. This alteration or destruction of structure is effected by 'ulcerative absorption,' or caries of the vertebræ; by 'progressive absorption' of the vertebræ, without caries or formation of pus; by an irregular growth of bone, whether combined with progressive absorption or not; or by either an ulcerative or progressive absorption of the intervertebral cartilages. To effect permanent distortion, this alteration of structure must produce such disproportions in the mechanism of the spine, as not only occasion a deflection from the upright attitude, but are incompatible with its retaining it, and render it impossible to place or restore the spinal column, at once or at will, to its perpendicular state, or to its spinal line or natural axis, either by the powers of volition, or by any mechanical power." 69.

Mr. Bampfield recurs to the old exploded doctrine of *unequal growth*, or malformation of bone, assumed by Glisson and others, as an efficient cause of distortion—" a doctrine," says Mr. B. " exploded too, against the evidence of the most perfect of our senses; for, in the anatomical museums, specimens of deformity arising from the unequal and irregular growth of bones, and of course of the vertebræ, may be seen; in some of which, the malformation or inequality is produced by diseased actions, but in others, simply by an exuberance of the '*formative principle*,' or by an error of nature, in exciting a pruriency, or occasioning a deficiency of growth." 76.

When an increased growth of bone takes place on one side of the spinal column, (which is a flexible one) it must necessarily elevate the parts above it on the same side, and make them recline over to the opposite side, which is thinner, and which must now sustain the greater share of weight. One consequence of this must be that a greater pressure will be made by all the superincumbent weight on the thinner side, whilst it will be diminished on the side elevated by the increased growth.

" Now pressure is the strongest power we possess of producing local absorption, and, as increased growth of bone is a cause of a permanent nature, the pressure too becomes permanent, (except in particular positions of the body,) and occasions absorption of that part of the bone, and of the intervertebral cartilage unduly pressed upon. Thus, increased growth on one side, and progressive absorption occasioned by pressure on the opposite side, both conspire to the same end, and both operate in destroying the natural proportions of the vertebræ, and of deflecting the erect spine from its spinal line to the form of a curve; and, in the degree that increased growth takes place on one side, and increased pressure and absorption on the other, the greater or less must be the curvature." 77.

Mr. Bampfield thinks that the same explanation will apply to some cases of excurvation and angular projection of the spine. In the dissection of H. Pittman (a case recorded by Mr. B.) and in some anatomical preparations of curved spine, it was found that a morbid enlargement or increased growth of the transverse and spinous processes, or of the bony ridge of some vertebræ, occasionally obtained, and which must have produced effects similar to those abovementioned. In some constitutions this absorption will go on without inflammation or ulceration being excited by pressure:—at other times, in less fortunate constitutions, slow inflammation and ulceration will be either speedily or eventually produced—" and thus it is that pressure may induce both the ulcerative and progressive absorption; but the ulcerative is much less frequently induced by it than the progressive."

" From the facts adduced, and the illustrations offered in this chapter, I think it may be deduced, as proved;—that the erect attitude of the body depends upon the vertebræ and intervertebral cartilages being of equal thickness, and that it cannot be preserved without this just proportion. That the temporary flexions of the spine are performed or permitted by those cartilages yielding to pressure, so that their thickness becomes temporarily altered. And that all permanent curvatures of the spine are caused by, or result from, the bodies of the vertebræ and intervertebral cartilages being reduced to a cuneiform shape or entirely destroyed; in which disproportionate state of parts it would be impossible to place the spine in the perpendicular or erect attitude." 84.

*Prognosis.* This may be favourable, as far as regards life, where there is no scrofulous taint, and the disease is taken early. How far the vertebral column may be restored to its true spinal line, is another affair to be noticed afterwards. When the curvature is caused by caries, or gangrenous destruction of the intervertebral substance, the prognosis must be, of course, unfavourable—and the same, if the caries be combined with external abscess. When the curvature, however large, is caused by progressive absorption, the prognosis may be favourable—" unless the patient be subject to attacks of asthma, constant head-aches, or hectic fever." This last symptom is fatal. Paralysis of the lower extremities is a dangerous, but not always a fatal symptom. When spinal pain ceases, the digestive function improves, the patient acquires flesh and muscular power; while freedom from dyspnœa, epigastric pain and tightness obtains, we may prognosticate a speedy recovery, as well as a termination of the spinal disease. A perseverance in the proper measures will considerably, if not totally, remove the curvature of the spine.

*Diagnosis.* In this section our author calls the attention of his brethren to the establishment of some distinguishing symptom between curvature with and curvature without caries; also to consider what symptoms may awaken suspicion, and lead to an inspection of the vertebral column in the early stages of disease. The subject he considers as difficult, and not hitherto much elucidated, because there are not many symptoms that are peculiar.

"The corded tightness or sense of constriction across the epigastre is, perhaps, the only symptom peculiar to curvature of the dorsal vertebræ. Disordered respiration, with diminution of the muscular power of the back or extremities, or irregular actions or convulsions of the muscles of the latter, with a sense of weakness or weariness not easily accounted for from any other disease, should excite suspicion, and lead to an inspection of the back, as ought a constant habit of stooping forwards or leaning to one side. In the curvatures without inflammation or caries, there is very little accompanying pain, and no pain is, in general, stated to be felt, unless the affected vertebræ are pressed upon. Curvatures with caries are preceded and accompanied with more marks of inflammation, such as pain, slight attacks of fever, and constitutional disturbance.

"In curvatures without caries, the patients generally retain a disposition to sit up and walk about; in those with caries, exercise frequently occasions pain, uneasiness, and speedy exhaustion of muscular power. In curvatures without caries, the general health is not often very much impaired, however great the curvature; at least, there are no symptoms from which much danger is apprehended: this general rule has its exceptions, as I have seen two cases of excurvation without caries, attended with dyspepsia, loss of appetite, torpid liver and bowels, and general emaciation; and one of the two was affected with paraplegia and disordered kidneys. Even in these, however, the countenance was more expressive of languor and debility than of pain.

"In curvature with caries, the general health seems to be impaired from the first accession of the inflammatory stage, whether it be simple or scrofulous inflammation; there is more pain complained of, which the patient endeavours to relieve by assuming attitudes that take off particular pressure, as by leaning backwards, or to the one side or the other; the patient gradually falls away from the first, and looks pale and languid, even when his appetite is tolerable; scrofulous swelling of glands, or affections of the joints sometimes supervene; great mental irritability ensues; the muscles become soft and flabby; and when the anterior ligaments are ulcerated through, and pus or sanies escapes from the carious vertebræ or ulcerated or gangrenous intervertebral substance, the symptoms of hectic fever with cough supervene, and the patient is generally carried off by it, and a wasting diarrhœa, assisted sometimes by the discharge of pus emanating from the diseased vertebræ. This diagnostic is not very satisfactory, but the history of the general state of health accompanying curvatures, has often been the best guide in forming a diagnostic opinion, so much worse is it in curvatures with caries than without. The inflammation, ulceration, or gangrene of the intervertebral substance have appeared to me to be attended with more local pain and tenderness, and more general suffering than caries of the bony structure, and the ulceration of the anterior spinal ligament is sometimes longer deferred in the latter case, but, when induced, abscesses are the result, which generally terminate in hectic fever, emaciation and death.

"There are not any early symptoms which may be pronounced *certain* diagnostics of a predisposition to curvature, or of an incipient curvature; when, however, a patient complains of any pain in the course of the spine, attended with a sense of constriction across the epigastre, and an occasional feeling of numbness or twitching of the upper or lower extremities, and is relieved by bending backwards, or to one side, or by lying down; suspicion should be roused and the back should be inspected. If any disease should exist, the dorsal muscles are generally wasted; if any degree of curvature exists, the projecting spinous processes will demonstrate it; if of inflammation, pressure, and the application of the hot sponge will excite pain. In curvatures without inflammation or caries, the hot sponge as often affords ease or comfort as it produces any other sensation.

"In an incipient excurvation, the undue prominence of any of the vertebræ, or rather of their spinous processes, will be most displayed by bending the body forwards, in which position any unnatural projection becomes evident." 90.

*Treatment.* In respect to the prevention of spinal curvature, it is remarked by Mr. Bampfield that, where rheumatism, strains, or contusions are in operation towards the production of the disease, the recumbent posture will be the surest preventive—and the same may be said of malposition; but *then* the recumbency should be alternated with suitable exercise of the muscles. "Generally speaking, says he, in all cases of incipient curvature, the recumbent posture should be much adopted, with such a degree of exercise of the dorsal muscles, as each particular case will admit of."

Where inflammation is present, exercise, of course, is injurious. In short, the general indications may be stated as follows:—*first*, to give the vertebral column effectual relief from pressure—*secondly*, to secure the bodies of the affected vertebræ from the irritation of motion in the erect attitude—*thirdly*, to assist the vertebral column in regaining its true spinal line—*fourthly*, to obviate occasional symptoms, rectify disordered functions, and promote the general health.

The *first* indication or object is evidently to be sought for in the recumbent posture, either on the inclined or horizontal plane. As it requires no mathematical demonstration to prove that the superincumbent weight is more effectually taken off the spine by the latter than by the former, so the horizontal position is the one preferred.

The second indication is to be fulfilled by abstaining from all movements and positions, *whilst recumbent*, which throw weight on the bodies of the vertebræ. At the same time experience shews that different positions of the body can be adapted to different forms of curvature "with striking and peculiar benefit." Mr. Bampfield employs three positions of horizontal recumbency, viz. on the back, on the abdomen, and on one side.

*Excurvation.* The general indications are all to be observed in this variety of curvature. Our author's experience, he avers, has proved to him that the *facial horizontal position* possesses infinite superiority in this variety of curvature, and he is surprised that reasoning, *á priori*, had not sooner led to its adoption. As this is an original proposition of Mr. Bampfield's, we shall allow him to speak for himself.

"When any one in health is lying on the facial horizontal position on a soft medium, for instance, a feather bed, the vertebral column, partly by its absolute gravity, falls or inclines inwards more than in any other natural position of the body, and forms a graceful inward curve of the lumbar and lower dorsal vertebræ, and should the vertebræ just mentioned be those involved in the curvature or angular projection, (as they frequently are,) or should this portion of the spine entirely form either of those distortions, this position will of itself tend to diminish the curve, and restore the column to its spinal line. Should the greatest point of outward projection be in the lumbar vertebræ, the facial horizontal position puts the flexor muscles of the thigh on the stretch, which tends to draw the lumbar vertebræ inwards. By this position, the horizontal surfaces of the anterior portions of the vertebræ will be separated to the greatest possible distance, to be effected from particular positions, by which, pressure will be effectually taken off from those portions; irritation removed; and an opportunity for regeneration of bone and intervertebral substance, as well as of its expansion, be afforded. This position enables the patient to exercise the muscles of the back, by moving the head backwards and forwards, or in any direction, by a very limited motion of the upper vertebræ, and by playing with the legs, without counteracting the two first general indications, by creating any pressure on the bodies of the vertebræ, and thus renders exercise of the dorsal muscles compatible with rest in the recumbent posture. And as excurvation is often remotely caused by an atony of the dorsal muscles; as physiologists infer that the development of the power of muscles is in the ratio of their exercise, and as it is a pathological fact, that muscles sometimes waste and lose their strength from inaction during disease, the importance of the moderate exercise of the dorsal muscles is manifest.

"In this position, the patient has the free use of his arms, and can raise himself on his elbows, by which latter movement the scapulæ are pressed backwards from their new lateral situations, and directed to their natural site on the back, and during this position, if the head be bent backwards, the spinous processes, (separated in this complaint in the middle of the curve,) and the posterior portions of the vertebræ are pressed more closely on each other, by which the spinous processes are approximated, the curvature diminished, and the anterior horizontal surfaces of the vertebræ still more relieved from pressure by their further separation.

"In this position, extension of the column, by pulling at the arms, and legs, can be conveniently accomplished at all times, as well as pressure made on the projecting vertebræ. This position renders friction, percussion, and shampooing available with convenient facility, and the management of issues, blisters, and setons, less troublesome and painful.

"When this position has been observed about three months, it has followed in all cases, where the patient has not been pot-bellied, or abdomen corpulent, that the spinal line, which, previously to its use, was evidently posterior to the natural axis in almost the whole extent of the spine, is gradually altered, and transferred, so that the line of the lower dorsal and lumbar vertebræ become placed anterior to it, and when the patient assumes the erect attitude, the upper part of the body no longer stoops forward, but falls backwards, and the patient can walk erect, with the lower part of the spine inclining inwards." 99.

Our author remarks further that, if the ribs have assumed the oval form, so that the patient is what is called *chicken-breasted*, the sternum, while lying in the facial horizontal position, is pressed towards the vertebræ by the whole superincumbent weight of that part of the body, and the ribs consequently pressed out on their sides, which tends to reduce them from the oval to the circular form. Lastly, the patient is permitted to lie comfortably on a soft feather bed, where he can amuse himself, "and enjoy a considerable freedom of movement." Mr. Bampfield makes several objections to the dorsal position on an inclined or horizontal plane, among which is stated the pressure made by the said plane on the projecting part of the excurvation, which, in some instances in his practice, could not be borne. The weight of the viscera also is an objection, in his mind, to the dorsal position, especially where the anterior spinal ligament or anterior portion of the vertebral bodies is inflamed. When the patient lies immoveably fixed on the back, the dorsal muscles are entirely deprived of motion and exercise—and consequently of the means of acquiring any degree of the strength which exercise communicates. In this position, no friction, pressure, &c. can be applied to the spine. Many other pros and cons respecting the dorsal and ventral positions are stated by our author, for which we must refer to the work itself.

Mr. B. observes, that, after the facial horizontal position has been kept three months, in conjunction with proper mechanical means, it very commonly happens that the spinal line of the lumbar and lower dorsal vertebræ becomes transferred anteriorly to the natural axis of the body, and when the patient is placed in the erect attitude, he no longer stoops forward as he did previously. When this change takes place, the patient should lie alternately on the dorsal and facial horizontal positions, by which the column will soon regain its natural axis.

The recumbent positions above-mentioned will be materially aided, in some cases, by mechanical extension of the vertebræ —by occasional pressure on the outer line of curvature—and by the application of certain apparatus. It is evident, however, that when a portion of spine becomes anchylosed through the whole surfaces of the diseased vertebræ, or in its posterior part alone, all efforts to alter its form will fail, and would be improper—hence cases of many years' duration are incurable.

The mode of employing extension and pressure is various. In the incipient stage, extension may be made by machinery, while the patient lies in bed, and pressure may be applied by the hand covered with a glove. Friction, shampooing, or percussion, alternately or in succession, may be used for an hour, by the nurse or parent of the patient, before the professional visit of the surgeon.

"After friction, extension and pressure have been employed, the apparatus of a compress, a quilted pad, made of what ladies call wadding, covered with silk or cotton, and a shield placed over the curvature, and secured by a long, broad bandage, rolled round the thorax, should be applied, as the patient lies on the pillows, which is the most eligible method, or he must be raised on his knees and elbows, so as to form a space under him that will allow free liberty to pass the bandage with the hand:—The bandage should be rolled as much as possible during expiration, when the ribs are not distended or raised; because the bandage would be then firmer, and because the power of inspiration, by raising the ribs, would tighten the bandage on the projecting sternum and vertebræ, and be almost a natural means of mechanical pressure.—Should inflammation of the vertebræ be present, attended with pain, or if the mechanical operations excite pain, in any degree, much complained of, they must be suspended or desisted from, until the inflammation has subsided, and the operations cease to occasion pain." 112.

Experience, Mr. Bampfield avers, has fully convinced him that the "*indiscriminate* and general use of issues and setons, which ensued on the publication of Mr. Pott's essay, may be dispensed with—because the cases in which they are not necessary, are more numerous than those in which they are."—Until experience has laid down an unerring guide for their employment, Mr. Bampfield hazards a conjecture that issues and setons will be beneficial in the cases "where scrofula is the occasional cause, during the period of scrofulous inflammation, ulceration, or caries." They are not necessary, and may be injurious, where the curvature depends on progressive absorption. Not so in disproportionate growth of bone, and in excurvations of the spine. In these they are often useful, especially the latter, which are frequently attended with inflammation or caries. Upon the whole, of the two plans of Pott and Baynton, Mr. Bampfield has witnessed more and speedier recoveries from the former (issues and setons) than from the latter—motionless dorsal horizontal position. We now come to the medical treatment.

"In the incipient stage of curvature from muscular debility, especially in children, chalybeates have been prescribed with much good effect, with an occasional laxative of pulv. rhei. twice a week. In this stage, local cold bathing has been useful, by applying cold water with a sponge to the back, during winter, and by cold affusion in the summer, provided there is no diathesis to pulmonic complaints, which would interdict its use. To recover or ensure the general health, the plainest diet, at regular intervals, should be enjoined, and yet the appetite preserved by a well-managed variety of simple food. The digestive organs are frequently deranged, and dyspepsia, obstipatio, and pain in the epigastric region prevail. The digestive functions have been strengthened, as well as the whole system, by obviating costiveness with rhubarb, or a combination of p. rhei. pil. hydr. et pulv. ipecac., and the exhibition of chalybeates, or the stomach bitters, with soda, or other alkalies. The peculiar pain across the epigastre sometimes requires opium for its relief; this pain and screaming in sleep, I have known removed by occasional purgatives.

"When the action of the kidneys is morbid, as may be inferred from the urine being scanty and turbid, and depositing lateritious sediments, it has commonly been restored to health by the use of magn. carb. soda carbon., with stomachics and gentle aperients.

"The peculiar pain of the epigastre, nervous irritation, and irregular spasms, perhaps from the spinal nerves being somehow disordered, by the tortuous direction the spinal marrow is obliged to assume, have generally yielded to the above remedies, surgical and medical, as have also paralysis of the lower extremities and incontinence of urine. In paraplegia, friction on the skin, flexion, and extension of the limbs, and warmth, have been used in aid of the above treatment. The different pulmonic affections must be treated by the remedies ordinarily employed in asthma, pneumonia, and cough. It may be observed, that asthma sometimes induces general dropsy in a short time, with purple-coloured lips, cheeks, feet, &c. and is frequently fatal to those whose spines and chests are much distorted. The milder attacks of asthma are sometimes greatly relieved by the vapour bath, used with the other remedies. The observance of the facial horizontal position diminishes the frequency of recurrence of asthmatic paroxysms, and combined with the use of the bandage, has the most decidedly good effect in relieving harrassing dyspnœa. Scrofula must be combated by appropriate remedies adapted to the different stages and symptoms of disease. In the inflammatory stage, frequent local bleeding, blisters, and setons, have been found useful during any accession or return of pain, with very slight alteratives at night, and a gentle hydragogue aperient in the morning, continued for a long period; when abscesses, or ulcerative absorption or caries are induced, and accompanied by emaciation, the bitters, sulph. acid and cinchona have their advantages. I have seen cases of curvature of the spine, in which scrofulous ulcers and abscesses of the joints of the extremities have been formed, and in which tonics and alteratives have proved highly beneficial, as well as the sulphuric acid. When the bladder loses its expulsive power, the catheter must be used until it regains it." 118.

Under proper management the state of the curvature and of the general health often undergoes considerable amelioration in the course of a month. But the change from the constant use of the recumbent posture to that of erect attitude should be gradual, " and the treatment should be divided between exercise in the erect attitude, gradually increased, and rest."

"When the destruction of the horizontal surfaces, or bodies of the vertebræ, has been produced by progressive absorption, although the regeneration of bone may have been prevented for years, by the patient remaining subject to the effects of pressure and of motion, by continuing to sit up and walk about; yet, soon after the patient is confined to the proper horizontal posture, by which rest is secured and weight taken off, the formation of new bone begins, and proceeds both anteriorly and posteriorly in the following manner, if it be allowed to deduce such an explanation of the arrangement from the observations made on the appearances presented on dissection. In the posterior portions of the vertebræ, the connecting ligaments of the spinous processes are successively converted into cartilage and bone, as are sometimes those of the oblique processes. Anteriorly, if the bodies of the vertebræ have been removed by progressive absorption, which generally leaves the anterior spinal ligament entire, depositions of cellular fat (sometimes) and then of bony matter take place on the ligaments, which gradually increase, until they fill up the chasm formed between the remaining vertebræ, and become united to them, and, during this process, the ligament itself becomes ossified. When the horizontal surfaces only have been absorbed, and not the entire bodies, the formation of callus may ensue from the surfaces, and wholly fill up the chasm, provided all pressure be kept off and motion laid aside during its formation, as is done in cases of fractured bones, on the same principle of propriety and necessity. If the destruction have proceeded from caries, more particularly scrofulous caries, the perfect regeneration can seldom commence before the scrofulous action has ceased, and the spine is placed in a favouring situation; ossification also proceeds differently, for the vessels form granulations that become ossified from the surfaces of the bones, bounding the upper and lower part of the spinal chasm, which approximate, and by degrees consolidate, and produce a bony union." 123.

In this, as well as in other species of curvature, active exercises of the muscles situated on the posterior part of the trunk, and on the anterior parts of the thorax and neck, can be advantageously employed in the incipient stages of deformity, and in the advanced stages of recovery, when the disposition to disease is corrected, and the vertebræ are sufficiently firm to bear the superincumbent weight—provided always, there is no inflammation of the vertebral joints present. The modes of exercising the muscles have been already adverted to, while reviewing the various works on the spine, and need not here be repeated. For many judicious remarks on instruments, exercises, and apparatus, we refer the reader to Mr. Bampfield's work, from page 127 to page 156.

*Incurvations of the Spine.* These are less frequent than excurvations, and do not describe such large curves. They are rarely attended with such serious consequences as the other curvatures. Incurvation is formed by a gradual and regular bend inwards of the vertebræ affected, presenting the appearance called "*hollow backed*"—and dissection shews such vertebræ of a greater thickness on the anterior than on the posterior parts of their bodies.

"In the lumbar and lower dorsal situations, it is sometimes caused by the gradual contraction of the flexor muscles of the thigh, induced during the existence of lumbar or psoas abscess, for the patients so affected are almost sure to assume and indulge in the bent position of the thigh; or this position may be involuntary, and the contraction the pure effect of disease; in either case, the contraction draws the vertebræ inwards, (more particularly when the weight of the extremity is depending from the muscles,) and the thigh upwards, so that, in truth, the muscular contraction should be regarded as the primitive disease in those instances, and the incurvation as an effect. Lumbar incurvation sometimes arises from carrying very heavy weights on the head, or suspended from the neck. One extremity becoming shorter from diseased hip-joint has also led to this incurvation. It has also arisen from an increased and disproportionate growth of the anterior portions of the bodies of the vertebræ. The cervical incurvation is sometimes caused by large heads inclining backwards, and chiefly produces inconvenience and disorder by its pressure on the œsophagus, rendering deglutition difficult, and extending to the trachea, so as to affect respiration in some degree. It is also occasioned by rickets," 158.

*Treatment.* In all cases of incurvation, the dorsal horizontal position is considered by Mr. B. as the most proper. If it be in the cervical vertebræ, the chin should be drawn in towards the neck, which tends to throw the cervical vertebræ backwards. If in the loins, the flexion of the thighs tends to remove it—but this flexion should not be constantly employed.

*Lateral Curvature.* This disease, Mr. B. observes, almost universally occurs *during the growth of the body*, and is almost as common as excurvation. It rarely occurs in infancy or after the adult age. The first deviations of the spine to either side are seldom observed, and the first symptom that usually attracts attention is the elevation of one of the shoulders, or depression of the other. When noticed, from three to five of the spinous processes may be seen to form a gradual aud slight curve to one side, more especially when the patient is standing or sitting upright. This curve almost always begins in the middle of the vertebral column, bearing a resemblance to the italic *S*; and in this state can frequently be reduced to the spinal line by recumbent posture and extension. Symptoms of nervous irritation and derangement are far from common at the commencement of lateral curvature, "nor indeed do they constantly occur at any stage of the progress of the distortion." It is well known that one curvature produces another in the opposite direction, in consequence of the effort made to preserve the equilibrium of the body. Lateral curvatures are formed by slow degrees; but here, as in excurvations, if the patient be assailed by any acute disease, as a fever, or any of the exanthemata, inducing debility, the progress of the curvature is accelerated rapidly until the strength is regained.

"If an original disproportion of growth of the bones of the vertebræ do not primarily give rise to any lateral curvature, an artificial disproportion of the thickness of the sides of the vertebræ ensues as soon as any degree of curve is formed, and increases with its increase. The bodies of the vertebræ and the intervertebral substance are gradually reduced to a cuneiform shape in different degrees, and are consequently diminished in thickness on the concave side or inner line of the curves, and are, for the most part, preserved of their natural dimensions on the convex side or outer line of the curves, and the dimensions of the vertebræ, and intervertebral substance in the centre of those curves exhibit the greatest disproportion. The middle or dorsal curve includes about eight of the upper dorsal vertebræ, and sometimes the whole; the upper or cervical curve is generally formed by the cervical vertebræ alone; the lumbar or lower curve as frequently implicates three, four, or the whole of the lumbar, and about four of the lower dorsal vertebræ.

"In some cases, the two lower lumbar, and some of the cervical vertebræ maintain their spinal line. In the advanced stages of lateral curvature, the distortion is not confined to a mere inclination of the vertebræ to the sides as the name implies, for the dorsal portions of the curved spine take a tortuous or serpentine direction, by which its spinous processes, instead of projecting directly backwards as usual, are twisted to one side, and are directed more to the concavity of the curve, than the convexity.* The effects of this spiral turn of the dorsal vertebræ are remarkable, and greatly contribute to increase the deformity; for, by it, the transverse processes and vertebral ends of the ribs are turned obliquely backwards on the convex side of the curve, and are advanced obliquely forwards on the other side; the ribs, on the side they are turned backwards, form a hump, projecting from half an inch to two inches and a half, posteriorly to the spinous processes of the dorsal vertebræ, and in the untoward turn and longer curve they are obliged to take to arrive at the sternum, they sometimes closely approximate or actually touch the lateral portions of the bodies of the vertebræ." 170.

Mr. B. avers that instances of carious vertebræ in this variety of curvature are very rare, and where they have occurred, "the caries has been occasioned by scrofulous action, which has extended to the intervertebral cartilages and ligaments, and excited an ulcerative action in them," inducing the necessity for anchylosis, as in excurvation. In the generality of cases, he observes, the integrity of the joints is preserved, and their articulations are so distinctly maintained, as to allow of considerable motion in all directions, with the body bent to one side, or curved in different directions to both. These lateral distortions necessarily occasion considerable changes and derangements in the muscles situated on the trunk. From the shortening of the ribs, and from their being situated in closer contact, or actually anchylosed on the concave side of the curvature, the capacity of the thorax on that side is diminished, and the lung it incloses is necessarily prevented from developing itself and arriving at its natural size, whilst the lung on the convex side of the curvature is frequently much compressed by the too close and unusual approximation of the ribs to the bodies of the vertebræ on that side. Hence a free expansion of the lungs in inspiration cannot be effected, and the patient suffers

* "Dr Dods has recently devoted a volume to this variety of lateral curvature under the name of rotated or contorted spine."

from difficult respiration, and disordered pulmonary circulation. The calibre of the spinal canal is frequently contracted about the centre of the curves, and is sometimes diminished to one half of the natural size. Although diseased action is not frequently set up in the medulla spinalis or its membranes, yet irritation is sometimes propagated to them from the bones, when they are carious, or otherwise diseased.

"The medulla spinalis is necessarily diminished in its volume, in that part of it which occupies those portions of the spinal canal lessened in its calibre. Its fibres are not of one uniform length, as they must be longer on the convex side of the curve than on the concave. The nerves proceeding from the medulla spinalis are sometimes smaller, even by one half on the concave side of the curves than on the convex, and hence it may be fairly inferred, that the organs, bones and muscles, deriving their nervous influence and energy from such diminutive nerves must be deficient in both, and the actions which depend upon them must be defective. The nerves must take unnatural courses, and be shorter or longer than natural, as they happen to proceed from the concave or convex side of the curves.

"In the generality of cases of lateral curvature, there is not any pain complained of in the course of the curves, nor is any felt or excited by pressure; nor is the power of the muscles so much weakened in general, as in excurvation. Yet, when scrofulous irritation and inflammation, with the results described in the last paragraph, are experienced by the bones and medulla spinalis, pain is felt in the course of the curvature; spasms of the extremities and diaphragm take place; the muscles of the extremities become disobedient to the will, and the patient cannot walk steady, or the muscles may become paralytic;* obstinate constipation ensues; incontinence of urine and fæces casually happens; dyspnœa with a sense of suffocation occurs; the irritation of the medulla spinalis is communicated to the nerves united to and proceeding from it, and various nervous symptoms and feelings are induced; in young females, hysteria sometimes takes place, and the menstruation is painful or suppressed;—this latter event is, however, more common in excurvations, as should have been observed. It may be stated, that Mr. Lloyd relates a case of lateral curvature attended with lumbar abscess.—*p.* 258, on *Scrofula.* This is an uncommon occurrence." 178.

In numerous cases of lateral curvature the general health is not much impaired, during the formation of the curvature, unless scrofula be present. Permanent lateral curvature, however, stunts the growth, especially of the lower extremities, probably from the diminution of the nerves proceeding from the

* "It has been observed by many authors, that the condition of the muscles in the paralysis from diseased Spine, differs from that in ordinary palsy, and that the muscles are not so soft, but the joints are more rigid."

spinal marrow. In the progress of this complaint, some degree of *excurvation* occasionally arises.

"The remote Causes of Lateral Curvature are rachitis, or disproportion of growth of the sides of the vertebræ; unequal length of the lower extremities; undue pressure on one side of the vertebral column from carrying heavy weights on one arm, by which the spine is bent to one side; an inclined position of the body to one side long continued; muscular debility; rheumatism of the intercostal and dorsal muscles; abscess on the side of the neck or trunk; scrofula; tumors. Is unequal action of the dorsal muscles a cause?" 180.

As Mr. Bampfield's experience in lateral curvature is extensive, he considers himself justified in inferring that RACHITIS is the fertile source of them in two ways:—*First*, by creating an irregular growth of the bodies of the vertebræ, which destroys their natural and relative proportions—and *secondly*, by occasioning a disproportionate growth of the lower extremities, which varies or destroys the natural centre of gravity of the body. "In most instances of spinal distortion from this cause, unequivocal appearances of rickety growth and deformity have displayed themselves in the bones of the upper or lower extremities." Mr. Bampfield has seen some instances of incipient lateral curvature produced in young females who were employed, during their growth, in carrying and nursing children, when their strength was inadequate to the burthen, the weight of the child compelling them to bend. This, he thinks, is not in favour, however, of the tenets of some recent authors, "that an inequality in the bulk of the arms, and of one side of the body, combined with the more constant use of one arm, will produce lateral curvature, by drawing the body down on one side, which causes other portions of the column to be bent in the opposite direction, to preserve its equilibrium." Mr. Bampfield has seen instances of lateral curvature produced by a habit of long-continued inclination of the body to one side, after the adult age, in insane persons. In the young and growing this is a more common event. Some eminent artists in London are distorted from this cause—malposition.

"Young ladies and boys who sleep two in a bed are apt to lie always on the same side of the body and bed, and become liable to lateral curvature from this cause, as are all young persons who sleep constantly on one side, with the side of the head and shoulder resting on pillows, as in that position, the spinal column is bent into a lateral curve. Young persons who always sit on the same side of a fire or window, are also liable, by acquiring a habit of leaning to one side." 184.

Of all the causes of lateral curvature, Mr. B. observes, none has been so generally and popularly received as—"the unequal action of the dorsal muscles." He thinks the abettors of this doctrine have assumed the facts without any proof. No explanation has yet carried conviction to his mind. It cannot, he avers, be demonstrated that an unequal power exists in the dorsal muscles. The circumstance of there being double or triple lateral curvatures, in different directions, is adduced by our author, as a proof that this inequality of power in the long muscles of the back is a chimera of the imagination.

"I have observed the condition and action of the dorsal muscles in the earliest stages of lateral curvature, attended with muscular debility; and the following were the phenomena they represented. After the patient has kept the spine erect, until the muscles are fatigued, the spinal column suddenly sinks down, and bends to one side, and the patient inclines to the other, to maintain the balance. In this state, they remain quiescent." 187.

Mr. Bampfield accuses some fashionable follies of modern education as justly responsible for this species of curvature, especially in young females—first, by establishing habits that deprive the muscles of the back of their natural exercise and actions, thus preventing the attainment of their natural strength—secondly, whilst in this state of debility, by endeavouring to subject the spinal muscles to more exertion than they are capable of using, in the injunctions they receive to keep the body constantly erect in standing or sitting attitudes.

"Thus the spinal muscles of young females are doomed to inaction, by the trunks of their bodies being imprisoned in stiff stays, or their movements abridged and confined by the use of collars, braces, back-boards, or by being stretched motionless on reclining boards, or school-room floors;—or they are subjected to long continued exertion, and the use of one posture, which all our muscles abhor, and soon become weary of, by being placed in education chairs or stools, the long forms of school-rooms, or on the round stool, to practise for hours on the pianoforte or harp, with strict injunctions to keep the body quite upright, and menaces of punishment, if they stoop or bend in the least; but the muscles must sometimes obey divine, instead of human laws, and when fatigued and weary in the erect posture, must gradually follow the Creator's law, and seek repose, by allowing the body to sink into an inclination to one side or the other, and, by laying the basis of lateral curvature, produce the reverse of what human wisdom intended." 188.

We fear Mr Bampfield's strictures on the management of schools are too severe—and his plans of improvement, somewhat Utopian.

"Without digressing further, I would observe, instead of stiff stays, back boards, reclining boards, education chairs without backs, military marching, &c. let the boys and girls have *no clothes* or apparatus to limit their movements, and when weary, let them sit down on chairs with proper curved backs to support the spine, or lie down for rest, or, in fact, seek repose as they find most agreeable, when they are fatigued, or can no longer maintain an erect attitude conveniently. Let the girls have *a large field* or play-ground, let the boys, also, have *the range of the country*, within the sound of the school-bell. Let the girls engage in the games of battledore and shuttlecock, skipping, dancing, and *all that they can play at*. Let the boys play at cricket, trap and ball, shinney, fives, skipping, running, coits, marbles, climbing trees, jumping, &c. and we shall not have many distortions of the spine; and, without intending to give offence, I will venture to express an opinion, that if the amount of a captain's pay was laid out for the use of the boys, at the military asylum, in the purchase of cricket bats, trap and balls, skipping ropes, swings, shinney sticks, marbles, coits, &c. there would not be any occasion for exercise masters, or surgeons to cure deformities, unless arising from scrofula or accident." 191.

The *immediate* causes of lateral curvature are the same as of permanent spinal distortion in general—" and will be found in the morbid alteration of structure of the sides of the spinal column, producing such a disproportion between them, as renders it impossible to restore it at once to perpendicular figure, and retain it in its true spinal line." This alteration, he thinks, is rarely effected by scrofulous caries, or ulcerative absorption on one side of the bodies of the vertebræ—and when it is produced thus, the curvature is generally single, and the scrofulous caries only affects one or two vertebræ. Scrofulous inflammation and ulceration, as was before observed, generally attacks the whole horizontal surfaces of the vertebræ, and produces *excurvation*, to which some lateral curvature is occasionally added. " The ordinary lateral curvature (to which the one just mentioned is a rare exception) affects many vertebræ, and is produced by progressive absorption, or by an undue growth of bone combined with it." The same mode of explanation is here used by Mr. Bampfield as was made when treating of excurvation. It has happened in his own practice, however, "that more cases of lateral curvature have originated in a disproportionate growth and length of the lower extremities than from any other single cause." This circumstance, he thinks, has not been sufficiently noticed by surgeons. The disproportion varies from half an inch to two inches and a half, and arises, he imagines, either from an extraordinary development and growth of one extremity, the diminutive growth of the other extremity—or from the ancle or knee joints bending, so as to cause one to be shorter than the other. The former is the more common cause, as the latter frequently exists without producing curvature. The *modus agendi* of these causes is so obvious as not to require a word. Indeed the principal fault which we have to find with Mr. Bampfield's work is the unnecessary explanations so often and so fully given.

"Patients affected with lateral curvature seldom lose their lives, even when the disease has been left to nature, unless scrofulously affected. It, however, permanently occasions dyspnœa and asthma, and the health of the subjects of this curvature is generally delicate, and easily disordered, but there are many exceptions to this observation, and, aided by a guarded regimen and regular habits, a good share of health may be enjoyed by those doomed to endure for ever this deranged structure." 199.

The section on "the dimensions of the vertebræ and ribs, in some specimens of lateral curvature," we must pass over, as it is illustrated by figures and wood-cuts, which must be seen with the eye to be understood.

*Treatment of Lateral Curvature.* Our author comprises under two heads the consideration of this important subject. 1st. Where the curvature arises from disproportionate length of the lower extremities—and 2dly, where it arises from any other cause except this. In the first case, it is evident the cause must be removed before the effect can be expected to cease. The treatment in these cases must consist, principally, in the adaptation of mechanical apparatus to equalize the positions and movements of the body. These need not be explained. Exercise and rest alternately, with tonics and regulation of the digestive organs, must be resorted to as auxiliaries to the apparatus.

Issues or setons can be of no use in this species of curvature. When the curvature has arisen from an *irregular and disproportionate growth of the vertebræ*, "which rickets so frequently disposes them to assume," our author has attempted to remedy the incipient stages by the exercises so highly extolled by various authors;—but not with the success he expected—in fact, he says, they "have decidedly failed," so that he always adopts the recumbent posture, and denies the patient the privilege of sitting up, allowed in lateral curvatures from other causes.

"If then it be admitted that the comparative value of the modes of treatment be sometimes in favor of a particular recumbent posture, when the curvature arising from rickets is in the incipient state, and the spine only deviates from the straight line into the form of an Italic *ſ*, (s,) very little doubt will be entertained of its superior value and propriety, when the lateral curvature has been neglected or maltreated, from whatr ever cause it may arise, until it has assumed the form of the Greek *s*, sigma. For, in the latter cases, all the medical and mechanical means that reason can suggest and experience sanction, should be assiduously employed to obtain even a chance of complete success. Proper position, extension, pressure, friction, exercises in the recumbent posture, &c. are among the natural and mechanical aids. There are two positions for common use of almost equal advantage, and they are both obliquely lateral, on a horizontal plane or couch, made for the purpose, and covered with a mattress; hence, the patient should be directed, either to lie obliquely on the side on which the ribs protrude backwards, or, in plainer words, to lie on the hump, as the superincumbent weight of the body bearing on this projection, will be equivalent to a certain degree of constant pressure, that tends to press those ribs forwards, and to direct the convexity of the spine towards the spinal line; or, he should be enjoined to lie on the face with an oblique direction to the opposite side, so that a dosser of lead, modelled in some degree to the shape of the distorted ribs or hump, and varying in weight according to age, or a bag of shot that will assume the turn of the ribs, may be laid on the projection so as to maintain a constant pressure, with the same indications as in the last position." 220.

The spine should be kept as long and as often extended, by mechanical contrivances, or by persons pulling at the extremities, as the patient can conveniently endure. During this extension, the surgeon or some person under his direction should employ pressure with his hands on the curved portions of the spine and projections of the ribs, for half an hour or an hour every day.

"With his thumbs placed against the sides of the spinous processes, on the convex side of the curve, he can press the vertebræ towards the spinal line, and, by laying the palms of his hands on the backward costal projections, he can press them forwards, and, by giving a lateral direction to the pressure, cause the vertebral extremities of the ribs to aid in directing the distorted vertebræ to their natural axis. Where there are two or three curvatures, a person may act on each side." 221.

Friction and shampooing he has found profitable, and they may be applied at all convenient opportunities. The patient should not be allowed either to sit or stand upright, as it is necessary to free the spine from all superincumbent weight in these cases of ambilateral curvature, as it is in excurvation. "The particular horizontal postures recommended, should be constantly observed." It is some consolation that a variety of these postures is allowable. To prevent the spine from bending during sleep, the patient should wear a pad, or a pair of stays, at night,—"but the use of the steel bow does away with their necessity." Every kind of available exercise in the recumbent posture should be put in force.

"Dyspnœa is a troublesome symptom in the advanced stages of lateral or ambilateral curvatures, and continues during life, if the curves be not removed; the most effectual relief I have found for it, is lying in the oblique position on the projection I have described, which tends to enlarge the capacity of the thorax and the power of expansion of the lungs, and to obviate the compression the lungs are subject to from the backward projection of the ribs and their approximation to the vertebræ." 224.

For a variety of practical remarks and ingenious speculations respecting lateral curvature, we must refer to the volume itself, from page 225 to page 238. We shall extract one more passage, however, bearing on the subject of promised cures in old-established distortions, where the growth of the body is finished.

"The view I have taken of the subject is the following;—During the growth of the body, parts of the bony structure that are deficient may be supplied, and those that are redundant and disproportionate may be absorbed, and the equality of size and proportion be restored. After the growth of the body has been completed, and the curvatures are attended with unequal proportions of the thickness of the sides of the vertebræ, and of the length of the ribs and extremities; it is clear that those alterations and additions of structure, necessary to restore symmetry of form, cannot be accomplished, without the creation of a new and successive growth of the diminutive bones or portions of bone; and, as the power of such a new creation is an attribute of the Deity, who has ended his work, and does not belong to man, it is impossible to remedy the finished deformity, or materially change it; yet, there are those who impiously promise and audaciously and ignorantly attempt this new creation, and who rob their fellow creatures of their liberty and time, by consigning them to a long and useless confinement, a motionless rest, and the stretching of machinery, in vain, to counteract the ordinations of Providence, that has given all things their bounds, and has said, thus far can ye go and no farther." 239.

*Angular Projection.* We shall not be able to dedicate so much space to this, the 9th Chapter of our author's work, as the subject deserves, in consequence of our closing limits. It is generally the 3d or 4th spinous process of the lumbar vertebræ which forms the prominent angular point in this disease. It has occurred in Mr. Bampfield's practice as frequently as either lateral curvature or excurvation. It most commonly takes place in young persons, between the age of three and twelve years.

"The projection is formed in a very gradual manner; the spinous process of the 3d or 4th lumbar vertebra is first observed to protrude a little, or both may protrude when first noticed; and as this projection increases and enlarges, it bears out from the spinal line, and elevates five or six of the spinous processes of the vertebræ above it, so that when the projection has made considerable progress, the lower spinous process projects beyond the spinal line from half an inch to nearly an inch, whilst five or six above are gradually inclined outwards from one of the dorsal vertebræ in its natural situation, to the extreme point of angular projection, between which and the lumbar vertebræ, *in situ*, below it, there is a space in which the fore-finger may be pressed.

"The transverse processes of the same vertebra generally project on each side of the spinous processes, and elevate and stretch the muscles and integuments, so as to produce a bow or arch from side to side, which is supported in the middle by the spinous process. The sternum sometimes projects a little, as in excurvation. In the majority of cases, the muscular powers of the back and of the lower extremities are gradually weakened; but in some cases no loss of power is evident. The patients in general become debilitated and soon fatigued from exercise, so that they cannot walk long without seeking a resting place to sit or lie down; when the projection is half an inch or more, the patient's body is commonly inclined forwards, the act of walking is imperfectly performed in a waddling gait, or the legs cross, and the patient falls down, or the dorsal muscles suddenly lose their power, and the patient stoops forward and seeks support by placing both hands above the knees. At all times, it is customary for the patients to avail themselves of any accidental support that falls in their route. In some cases, there is considerable pain experienced in the deranged vertebræ, and, in others, but very little or none, unless they are much pressed upon, and sometimes pain is not produced by pressure. The pains are sometimes sudden and severe, and compel the patients to throw themselves into the recumbent posture, wherever they happen to be when they supervene. Quick and sudden motions of the spine sometimes induce smart pains in the affected vertebræ. Pains of the back and of one or both knees, in some cases, disturb and prevent sleep, and compel the little patients to scream aloud. These pains generally occur in the night-time, and are often connected with, if not caused by, lumbar inflammation and abscess." 243.

Many cases of lumbar abscess are connected with this deformity. Dissection generally discloses disease of bone intervertebral cartilage and ligament. Sometimes on dissection, the vertebræ, cartilages, and ligaments are found sound in structure, "but an unequal or disproportionate growth of bone is evident, by which the spinous and transverse processes are thrown back, and form the angular point."

In respect to prognosis, our author observes that, every case of this disease which has been placed under his care, within six months after its formation, terminated successfully,—unless caused by lumbar abscess already produced.

“Cases of long duration, in which the previous symptoms of inflammation had existed, and in which emaciation, great debility, much pain of the back and hectic fever have been induced before due surgical attention has been paid, have terminated fatally.” 250.

If the lumbar pain abate—if the prevailing symptoms become mitigated—if the general health improve, the prognosis may be favourable, and *vice versâ.*

“The remote causes of this species of spinal distortion are contusions of the spine; sprains of the lumbar vertebral joints; muscular debility; pressure of the cyst of lumbar abscess; a rickety growth or malformation of the bony bridge; and, more frequently than any other, scrofulous or common inflammation of the intervertebral cartilage, and horizontal surfaces of the vertebral bodies.” 251.

The *immediate* causes of this complaint are, Mr. B. avers, the same as of other spinal distortions—“structural disproportion between the anterior and posterior portions of the bodies of the vertebræ or intervertebral cartilages, or destruction of the horizontal surfaces of one or more vertebræ.”

*Treatment.* Many of the indications of cure already pourtrayed for *excurvation* will apply here. Repose, in the facial horizontal position on a soft feather bed, is peculiarly advantageous, as, by this, all weight and pressure are taken off the anterior portions of the spinal column, and the progressive absorption suspended and prevented. Whenever symptoms of inflammation occur, or pain is felt on pressure or motion, topical bleeding, counter-irritation, “a daily hydragogue aperient, with alteratives,” a low regimen, and perfect quietude should be enjoined. Mr. Bampfield agrees with Mr. Brodie, Mr. Copeland, and others, that in all cases originating in intervertebral inflammation (which has always a strong tendency to ulceration) issues and setons are particularly beneficial.

“After the mechanical and medical means have been attentively employed about three months, if the patient be directed to assume the erect attitude, and the spine be examined, it will generally be perceived that the lumbar portion of the spinal line is inclined forwards; when this effect is produced, and towards the end of the cure generally, lying on the back, and the frequent exercise of the lower limbs in flexion and extension, will prove advantageous, as it will tend to bring the spinal line into the natural axis of the body, and the forcible extension of the flexor muscles of the thigh will draw in the lumbar vertebræ. In the most favorable cases, there remains an apparent projection of about ¼ of an inch of one or two spinous processes, or of one or two transverse processes, which protrude a little beyond the level of the spinous processes, but this does not alter the natural axis of the body, nor prevent the patient from standing upright, and preserving the erect attitude, and is a mark of some disproportion of growth still remaining," 260.

The last three chapters of Mr. Bampfield's work, on fractures, dislocations, inflammations, and other diseases of the spine and its contents, we must pass over—*first*, because we have already treated of those subjects in our review of M. Olivier's work in the first number of this Journal—and *secondly*, because we shall shortly again have the same subject on the tapis, in professed works on those affections. On these accounts, we must now draw our analysis to a close, without touching on the Appendix of Cases in illustration of the general principles laid down in the body of the work.

As Mr. Bampfield has gone to work in a very systematic manner, availing himself of the writings of others as well as his own personal experience, he has constructed an Essay that will perhaps prove more generally useful and acceptable to the practitioner than any single work on the same subject. For certain peculiar opinions and practices, which the reader must have observed in the course of this analysis, Mr. Bampfield is answerable, and will doubtless be able and ready to reply to such objections or criticisms as his professed *spinal* cotemporaries may think proper to put forth. He has freely criticised and commented on the writings and practices of others—he must expect returns in kind. With these matters, however, we have little or nothing to do. *Criticism* is of so very little consequence, in our opinion, that we are quite indifferent whether or not it enters into the composition of the Journal at all. This, to *a few* of our readers, may seem "passing strange;" but they will probably, on reflection, give us credit for having some little knowledge of what is most advantageous to the public as well as to ourselves. Without professing, therefore, to be initiated farther than our neighbours in the "craft and mystery" of spinal diseases, we can only say, in conclusion, that Mr. Bampfield's Essay appears to us to have *deserved* the prize conferred on it by the Medical Society of London—and this, we think, is commendation enough.

